# Human CD34^+^ Progenitor Cells Freshly Isolated from Umbilical Cord Blood Attenuate Inflammatory Lung Injury following LPS Challenge

**DOI:** 10.1371/journal.pone.0088814

**Published:** 2014-02-18

**Authors:** Xiaojia Huang, Kai Sun, Yidan D. Zhao, Stephen M. Vogel, Yuanling Song, Nadim Mahmud, You-Yang Zhao

**Affiliations:** 1 Department of Pharmacology, University of Illinois College of Medicine, Chicago, Illinois, United States of America; 2 Center for Lung and Vascular Biology, University of Illinois College of Medicine, Chicago, Illinois, United States of America; 3 Department of Pharmacology, School of Medical Sciences and Laboratory Medicine, Jiangsu University, Zhenjiang, China; 4 Department of Pulmonary Medicine, Zhongshan Hospital, Fudan University, Shanghai, China; 5 Department of Medicine, University of Illinois College of Medicine, Chicago, Illinois, United States of America; Chinese Academy of Sciences, China

## Abstract

Adult stem cell-based therapy is a promising novel approach for treatment of acute lung injury. Here we investigated the therapeutic potential of freshly isolated human umbilical cord blood CD34^+^ progenitor cells (fCB-CD34^+^ cells) in a mouse model of acute lung injury. At 3 h post-lipopolysaccharide (LPS) challenge, fCB-CD34^+^ cells were transplanted i.v. to mice while CD34^−^ cells or PBS were administered as controls in separate cohorts of mice. We observed that fCB-CD34^+^ cell treatment inhibited lung vascular injury evident by decreased lung vascular permeability. In contrast, CD34^−^ cells had no effects on lung vascular injury. Lung inflammation determined by myeloperoxidase activity, neutrophil sequestration and expression of pro-inflammatory mediators was attenuated in fCB-CD34^+^ cell-treated mice at 26 h post-LPS challenge compared to PBS or CD34^−^ cell-treated controls. Importantly, lung inflammation in fCB-CD34^+^ cell-treated mice was returned to normal levels as seen in basal mice at 52 h post-LPS challenge whereas PBS or CD34^−^ cell-treated control mice exhibited persistent lung inflammation. Accordingly, fCB-CD34^+^ cell-treated mice exhibited a marked increase of survival rate. Employing *in vivo* 5-bromo-2′-deoxyuridine incorporation assay, we found a drastic induction of lung endothelial proliferation in fCB-CD34^+^ cell-treated mice at 52 h post-LPS compared to PBS or CD34^−^ cell-treated controls, which contributed to restoration of vascular integrity and thereby inhibition of lung inflammation. Taken together, these data have demonstrated the protective effects of fCB-CD34^+^ cell on acute lung injury induced by LPS challenge, suggesting fCB-CD34^+^ cells are an important source of stem cells for the treatment of acute lung injury.

## Introduction

Acute lung injury (ALI) and acute respiratory distress syndrome (ARDS) remain one of the most common causes of acute respiratory failure in critically ill patients [1]. Despite recent strides in the understanding of the pathogenesis of ALI/ARDS, current treatments are mainly supportive with lung protective ventilation and a fluid conservative strategy and the mortality rate remains as high as 40% [1]–[4]. Thus, novel therapeutic strategies are needed to improve the outcome of this devastating disease. Recently, human mesenchymal stem or stromal cells (MSC) are shown to be protective in animal models of ALI induced by lipopolysaccharide (LPS) [5]–[8], live bacteria [9], [10], polymicrobial sepsis [8], [11], and pneumonia [10] as well as in an ex vivo perfused human lung injury model challenged with E. coli [12], [13]. These studies suggest that human adult stem cells is a potential therapeutic approach for the treatment of ALI and ARDS [14], [15].

CD34 is a member of a family of single-pass transmembrane sialomucin proteins [16]. Cells expressing CD34 (CD34^+^ cells) are normally found in the umbilical cord blood and bone marrow as hematopoietic stem cells, endothelial progenitor cells as well as activated endothelial cells of blood vessels [17]–[21]. CD34^+^ hematopoietic progenitor cells are a well-characterized population of stem cells that have been used clinically to reconstitute the hematopoietic system after irradiation or chemotherapy. Since the finding of putative human endothelial progenitor cells, a subpopulation of CD34^+^ mononuclear blood cells isolated from human peripheral blood induce angiogenesis [21], human CD34^+^ progenitor cells isolated from bone marrow, peripheral blood and cord blood have been tested in many preclinical models of ischemic vascular diseases. These cells are efficient to promote angiogenesis and provide beneficial effects on myocardial infarction [22], [23], peripheral ischemia [24], [25], and ischemic stroke [26], [27]. More importantly, some clinical studies also confirmed the beneficial effects of human CD34^+^ progenitor cells on coronary heart disease [28], [29]. However, little is known about the effects of human CD34^+^ progenitor cells isolated from umbilical cord blood on acute lung injury induced by sepsis. In addition, all the published results in preclinical animal models of ALI have been using cultured cells including human MSC. It is unclear whether the beneficial effects of these cells are derived from *in vitro* culture. Here, we show intravenous administration of CD34^+^ progenitor cells freshly isolated from human umbilical cord blood (fCB-CD34^+^ cells) attenuate LPS-induced lung injury in mice and promote endothelial cell proliferation responsible for restoration of vascular integrity following LPS challenge. To address the therapeutic potential of these cells, we employed fCB-CD34^+^ cells after establishment of lung injury at 3 h post-LPS challenge. These results suggest human fCB-CD34^+^ cells represent a novel therapeutic approach to inhibit inflammatory lung injury and promote vascular repair for the treatment of ALI/ARDS.

## Methods

### Ethics statement

Human umbilical cord blood was obtained from the New York Blood Center following the guidelines established by the University of Illinois at Chicago Institutional Review Board. All animal experiments were performed in accordance with protocols approved by the University of Illinois at Chicago Animal Care and Use Committee.

### Isolation of human umbilical cord blood CD34^+^ progenitor cells

Human umbilical cord blood was obtained from the New York Blood Center (New York, NY) following the guidelines established by the University of Illinois at Chicago Institutional Review Board. Low density cord blood cells (<1.077 g/ml) were obtained by density centrifugation on Ficoll-Paque PLUS (GE Healthcare Bio-Sciences AB, Uppsala, Sweden), from which CD34^+^ cells were isolated by the MACS CD34 progenitor isolation kit using immunomagnetic beads (Miltenyi Biotech, Inc., Auburn, CA) as described previously [30]. The purity of isolated CD34^+^ cells routinely ranged 90–99%. Purified CD34^+^ cells were resuspended in PBS and kept on ice overnight for transplantation next day. CD34^+^ cell-depleted cells (i.e. CD34^−^ cells) were used as controls.

### Mice

NOD.CB17-*Prkdc^scid^*/J (SCID) mice were purchased from the Jackson laboratory (Bar Harbor, ME). All mice (males, 3 month old) were maintained in the Association for Assessment and Accreditation of Laboratory Animal Institute of Health guidelines. All animal experiments were performed in accordance with protocols approved by the University of Illinois at Chicago Animal Care and Use Committee.

For survival study, mice following LPS challenge had normal access for water and food, and were monitored four times a day over the course of 7 days. Moribund animals were identified by labored breathing pattern defined as a decreasing rate of respiration and/or an inability to ambulate in response to stimulation. Moribund mice were euthanatized using CO2 followed by cervical dislocation (5 out of 18 nonsurvival mice were euthanatized). At the end of the study (day 7), all the survived mice were euthanatized with CO2 followed by cervical dislocation.

### Induction of lung injury

Mice received a single dose (5 mg/kg BW) of LPS (*Escherichia coli* 0111:B4) (Sigma-Aldrich, St. Louis, MO) by i.p. injection. To minimize suffering of the animals, all mice were anesthetized with ketamine/xylazine (100/5 mg/kg BW, i.p.) prior to tissue collection and lung preparation. For survival study, mice were challenged with a single lethal dose of LPS (11 mg/kg), and monitored for 7 days.

### Cell Transplantation

At 3 h post-LPS challenge, cells (fCB-CD34^+^ or CD34^−^) were transplanted to mice at a dose of 0.2X10^6^ cells/mouse in 150 µl through retro-orbital injection. The same amount of PBS was injected to a control group of mice.

### Pulmonary microvascular permeability assay

Capillary filtration coefficient (*K*_f,c_) was measured to determine pulmonary microvascular permeability to liquid as described previously [31]. Briefly, after 30-minute equilibration perfusion, the outflow pressure was rapidly elevated by 10 cmH_2_O for 20 minutes and then returned to normal. The lung wet weight changed in a ramp-like fashion, showing net fluid extravasation. At the end of each experiment, lungs were dissected free of non-pulmonary tissue, and lung dry weight was determined. *K*_f,c_ (ml/min/cmH_2_O/dry lung weight) was calculated from the slope of the recorded weight change normalized to the pressure change and to lung dry weight.

### Myeloperoxidase assay

Lung tissues were homogenized in 5 mM phosphate buffer and then centrifuged at 15,000×g for 20 minutes at 4°C. The pellets were resuspended in phosphate buffer containing 0.5% hexadecyl trimethylammonium bromide and subjected to a cycle of freezing and thawing. Subsequently the pellets were homogenized and the homogenates were centrifuged again. The supernatants were assayed for MPO activity using kinetics readings for 3 minutes and absorbance was measured at 460 nm. The results were presented as ΔOD_460_/min/g lung tissue [32].

### Lung transvascular albumin flux assessment

Evans blue-conjugated albumin (EBA) extravasation assay was performed as described[33]. EBA at a dose of 20 mg/kg BW was retro-orbitally injected into mice 40 min before tissue collection. Lungs were perfused free of blood with PBS, blotted dry, weighed and snap frozen in liquid nitrogen. The right lung was homogenized in 1 ml PBS and incubated with 2 volumes of formamide at 60°C for 18 h. Then the homogenate was centrifuged at 10,000×g for 20 min. The optical density of the supernatant was determined at 620 nm and 740 nm. Extravasated EBA in lung homogenates was expressed as micrograms of Evans blue dye per g lung tissue.

### Cell proliferation assay

BrdU (75 mg/kg BW, Sigma-Aldrich) was intraperitoneally injected to mice 4 h prior to tissue collection. Mouse lung cryosections (6 µm thick) were stained with FITC-conjugated anti-BrdU antibody using the In Situ Cell Proliferation kit (Roche Diagnostics) [31] and nuclei were counterstained with DAPI. Anti-vWF (1∶300, Sigma) and anti-CD31 antibodies (1∶40, Abcam, Cambridge, MA) were used to identify endothelial cells.

### Quantitative RT-PCR analysis

Total RNA was isolated using an RNeasy Mini kit including DNase I digestion (Qiagen). Following reverse transcription, quantitative real-time PCR analysis was performed with a sequence detection system (ABI Prism 7000, Life Technologies) with SYBR Green master mix (Roche Diagnostics). The following primers were used for analysis: mouse ICAM-1, 5′-GTCTCGGAAGGGAGCCAAGTA-3′ and 5′-CTCGACGCCGCTCAGAAGAA-3′; mouse TNF-α, 5′-ATGCTGGGACAGTGACCTGG-3′ and 5′- CCTTGATGGTGGTGCATGAG-3′; mouse IL-6, 5′-TCCAGTTGCCTTCTTGGGACTG-3′ and 5′-AGCCTCCGACTTGTGAAGTGGT-3′; mouse iNOS, 5′-ACATCAGGTCGGCCATCACT-3′ and 5′-CGTACCGGATGAGCTGTGAATT-3′. Primers for mouse cyclophilin were published previously [32]. The mouse gene expression was normalized to mouse Cyclophillin.

### Histology

Lung tissues were fixed by 5 min instillation of 10% PBS-buffered formalin through trachea catheterization at a transpulmonary pressure of 15 cm H_2_O, and then overnight at 4°C with agitation. After paraffin processing, the tissues were cut into semi-thin 5 µm thick. Chloroacetate esterase staining was performed using a Naphthol AS-D Chloroacetate (Specific Esterase) kit (Sigma-Aldrich) which stains specifically polynuclear granulocytes.

### Statistical analysis

Data are expressed as mean ± SD. Differences between groups were examined for statistical significance using student *t*-test or *ANOVA* followed by Dunnett's Multiple Comparison except for statistical analysis in the mortality study, which was performed with the Peto-Peto-Wilcoxon test. *P*<0.05 denoted the presence of a statistically significant difference.

## Results

### Freshly isolated human CD34^+^ progenitor cells inhibit lung vascular injury following LPS challenge

**Figure 1:**
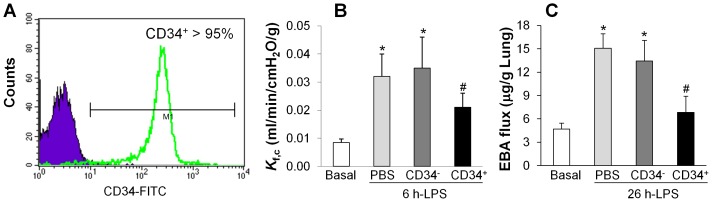
Inhibition of lung vascular permeability in mice treated with fCB-CD34^+^ cells. (**A**) FACS analysis demonstrating greater than 95% purity of CD34^+^ cells. Low density human cord blood cells (<1.077 g/ml) were obtained by density centrifugation on Ficoll-Paque PLUS, from which CD34^+^ cells were isolated by the MACS CD34 progenitor isolation kit using immunomagnetic beads. Purple histogram indicates matched isotype control and staining with CD34 monoclonal antibody is shown as green overlaid histogram. The purity of isolated CD34^+^ cells routinely ranged 90–99%. (**B**) Decreased lung vascular permeability to liquid in mice treated with fCB-CD34^+^ cells. At 3 h post-LPS challenge (5 mg/kg, i.p.), cells or PBS were administered and *K*_f,c_ was determined at 6 h post-LPS. Data are expressed as mean ± SD (*n* = 4/group). *, *P*<0.001 versus Basal; #, *P*<0.05 versus PBS or CD34^−^. (**C**) Lung vascular permeability assessed by EBA extravasation assay. At 26 h post-LPS challenge, mouse lung tissues were collected for EBA assay. Data are expressed as mean ± SD (*n* = 4). *, *P*<0.001 versus Basal; ^#^, *P*<0.01 versus PBS or CD34^−^.

Human CD34^+^ progenitor cells were isolated from human umbilical cord blood using the immunomagnetic beads with purity of routinely greater than 95% (**Fig. 1A**). To determine the effects of these freshly isolated CD34^+^ progenitor cells (fCB-CD34^+^ cells) on lung injury induced by LPS, fCB-CD34^+^ cells (0.2 X 10^6^ cells/mouse) were transplanted to SCID mice at 3 h post-LPS challenge. As comparison, same amount of CD34^−^ cells were transplanted to a separate cohort of mice. The same volume of PBS was administered as control.

lung vascular permeability was assessed by determinations of lung capillary filtration coefficient (*K*_f,c_) (permeability to liquid) [31] and EBA flux (permeability to protein) [33]. As shown in **Fig. 1B**, we observed a marked increase of *K*_f,c_ value, which normally peaked at 6 h post-LPS challenge in control PBS-treated mice at 6 h post-LPS challenge (5 mg/kg, i.p.). Intravenous administration of fCB-CD34^+^ cells decreased *K*_f,c_ value whereas human CD34^−^ cells did not show the protective effect. Similarly, fCB-CD34^+^ progenitor cells inhibited LPS-induced increase of EBA flux (which normally peaked at 24 h post-LPS challenge) in mouse lungs at 26 h post-LPS challenge (**Fig. 1C**). However, mice transplanted with CD34^−^ cells exhibited increased lung vascular permeability similar to PBS-treated controls.

### Inhibited lung inflammation in mice treated with human CD34^+^ progenitor cells

**Figure 2:**
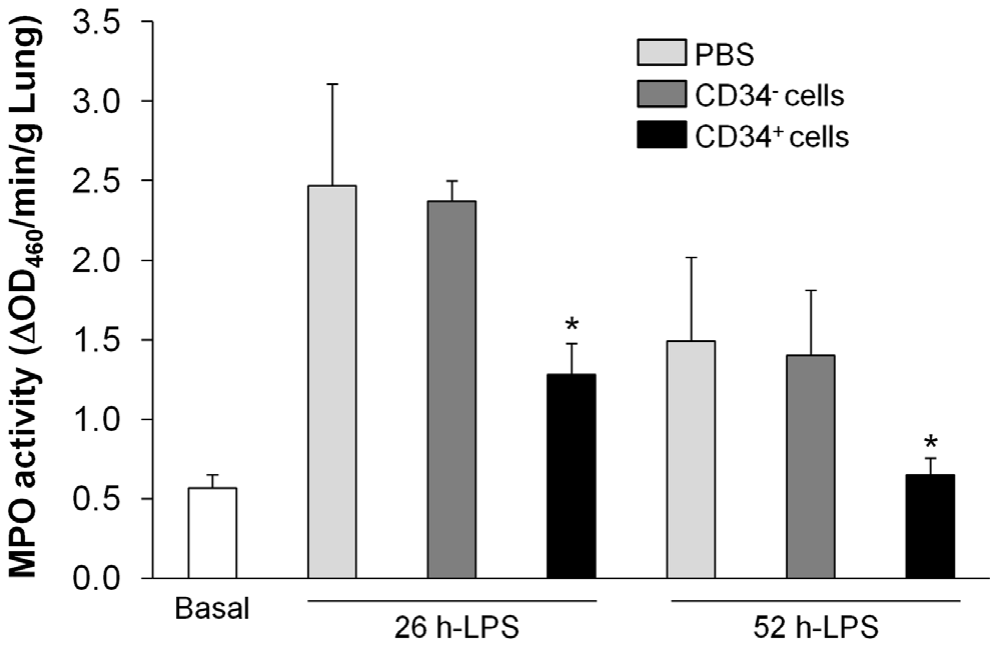
Inhibited lung inflammation in mice treated with fCB-CD34^+^ cells. Lung tissues at indicated times post-LPS challenge were collected for MPO activity determination. Data are expressed as mean ± SD (*n* = 4/group). *, *P*<0.05 versus PBS or CD34^−^.

We next assessed lung inflammation by measuring MPO activity, an indicator of neutrophil infiltration [31]–[33]. As shown in **Fig. 2**, at 26 h post-LPS challenge, lungs of mice treated with fCB-CD34^+^ exhibited lower MPO activity than those treated with either PBS or human CD34^−^ cells. At 52 h post-LPS challenge, MPO activity in lungs of CD34^+^ cell-treated mice returned to basal levels whereas it remained elevated in lungs of control mice treated with either PBS or CD34^−^ cells.

**Figure 3:**
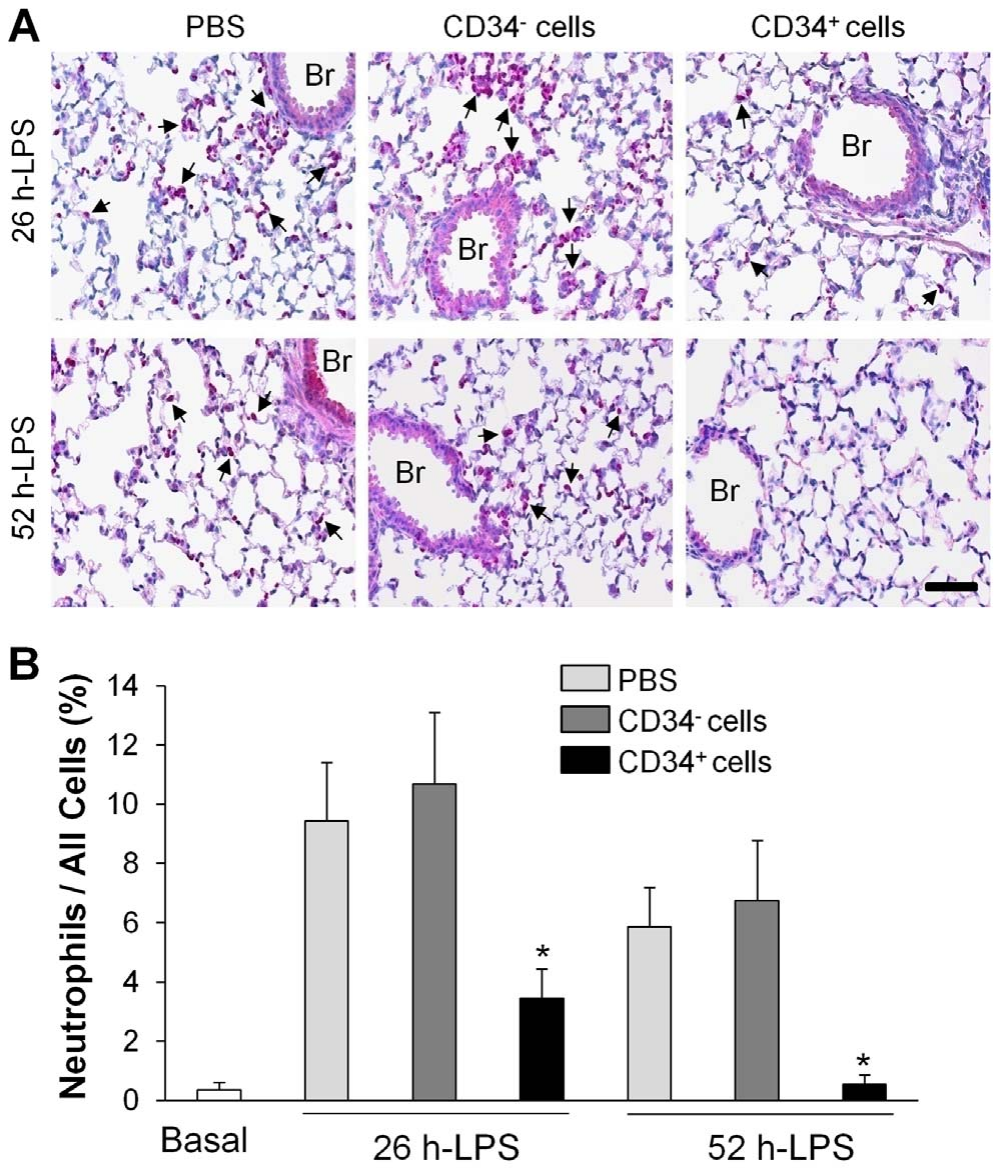
Decreased lung tissue neutrophil infiltration in fCB-CD34^+^ cell-treated mice. Lung tissues at indicated times post-LPS challenge were collected and sectioned for polynuclear granulocyte-specific chloroacetate esterase staining. (**A**) Representative micrographs of chloroacetate esterase staining (purple) showing neutrophil infiltration in mouse lungs after LPS challenge. Arrows indicate positive staining. Br, bronchia. Scale bar: 50 µm. (**B**) Quantitative analysis of infiltrating neutrophils in mouse lungs. Data are expressed as mean ± SD (*n* = 4/group). *, *P*<0.05 versus PBS or CD34^+^.

In addition, chloroacetate esterase staining, which stains granulocytic cells (mainly neutrophils) [34] revealed greater neutrophil sequestration in lungs treated with PBS or human CD34^−^ cells compared to lungs treated with fCB-CD34^+^ cells (**Fig. 3**). At 49 h post-cell transplantation (i.e. 52 h post-LPS challenge), only basal level of neutrophil sequestration was detected in mice treated with CD34^+^ progenitor cells.

**Figure 4:**
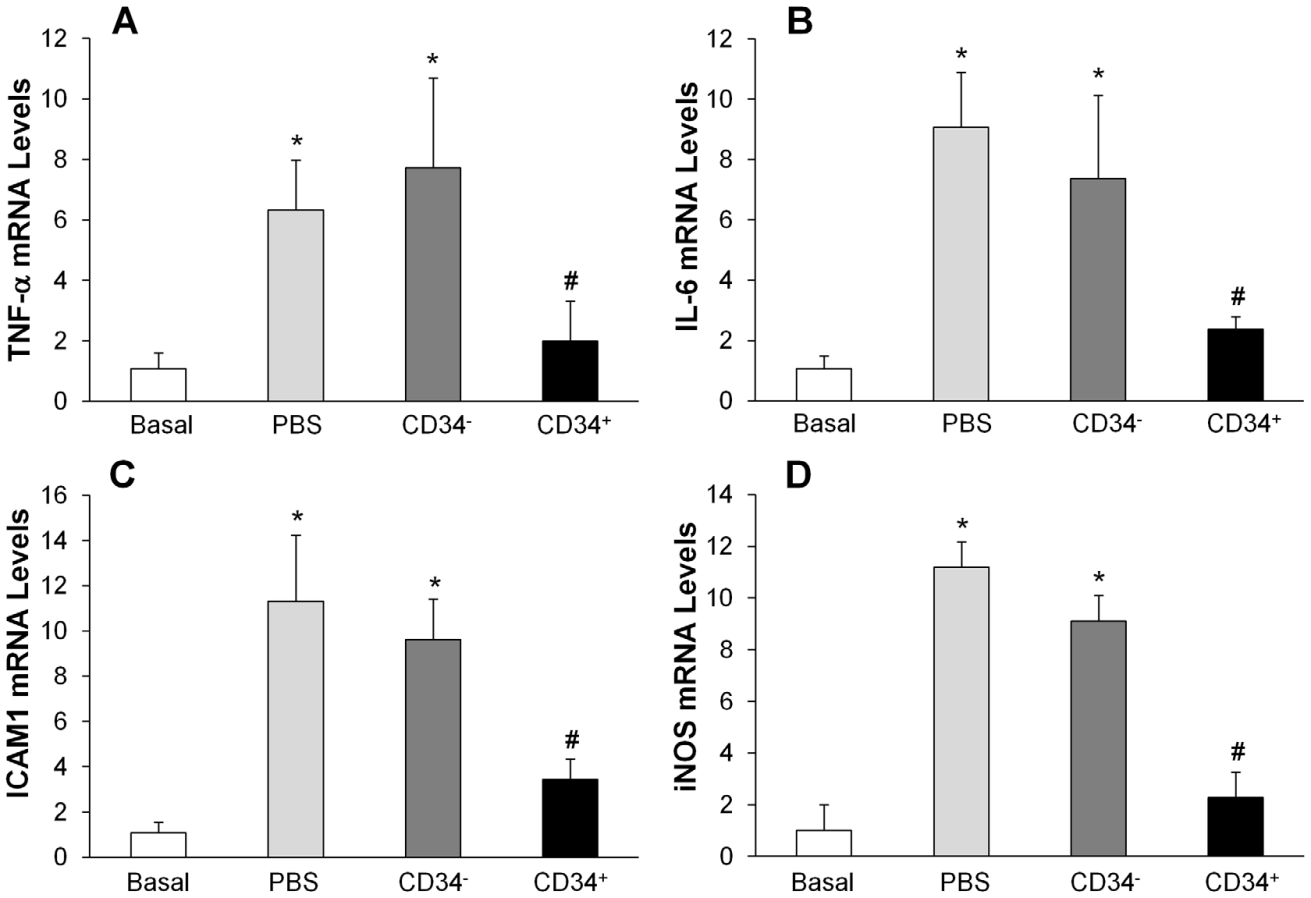
QRT-PCR analysis of expression of pro-inflammatory mediators in mouse lungs following LPS challenge. Lung tissues were collected at 26-LPS challenge for RNA isolation and QRT-PCR analysis. Data are expressed as mean ± SD (*n* = 4/group). *, *P*<0.001 versus Basal; #, *P*<0.05 versus PBS or CD34^−^.

Expression of proinflammatory molecules in mouse lungs was also quantified with quantitative real time RT-PCR analysis. As shown in **Fig. 4**, expression of TNF-α, IL-6, ICAM-1 and iNOS genes were markedly induced in the lungs of PBS-treated control mice at 26 h post-LPS challenge. fCB-CD34^+^ cell treatment resulted in a drastic decrease of expression of these proinflammatory molecules but CD34^−^ treatment had little effects.

### Marked increase of survival of mice treated with human CD34^+^ progenitor cells

**Figure 5:**
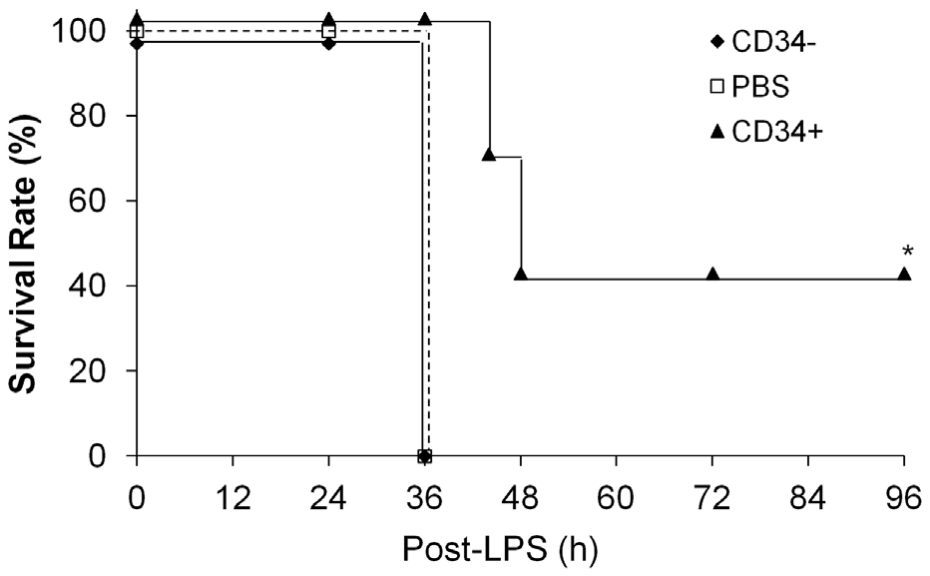
Increased survival of fCB-CD34^+^ cell-treated mice. After lethal dose of LPS challenge (11 mg/kg, i.p.), mice were monitored for 7 days to determine the survival rate (*n* = 7 per group). *, *P*<0.01 versus PBS or CD34^−^.

To determine whether fCB-CD34^+^ cell treatment promotes survival, mice were challenged with lethal dose of LPS (11 mg/kg, i.p.). As shown in **Fig. 5**, all PBS-treated control mice died within 36 h post-LPS challenge. Similarly, all CD34^−^ cell-treated mice also died within 36 h. At the same time period, all mice treated with fCB-CD34^+^ cells survived. By 48 h post-LPS challenge, approximately 60% of the fCB-CD34^+^-treated mice died. Intriguingly, the remaining 40% of these mice survived for at least one week and they recovered from LPS-induced injury and became active by the end of the study at 7 days post-LPS challenge.

### Marked induction of lung endothelial cell proliferation following human CD34^+^ cell treatment

**Figure 6:**
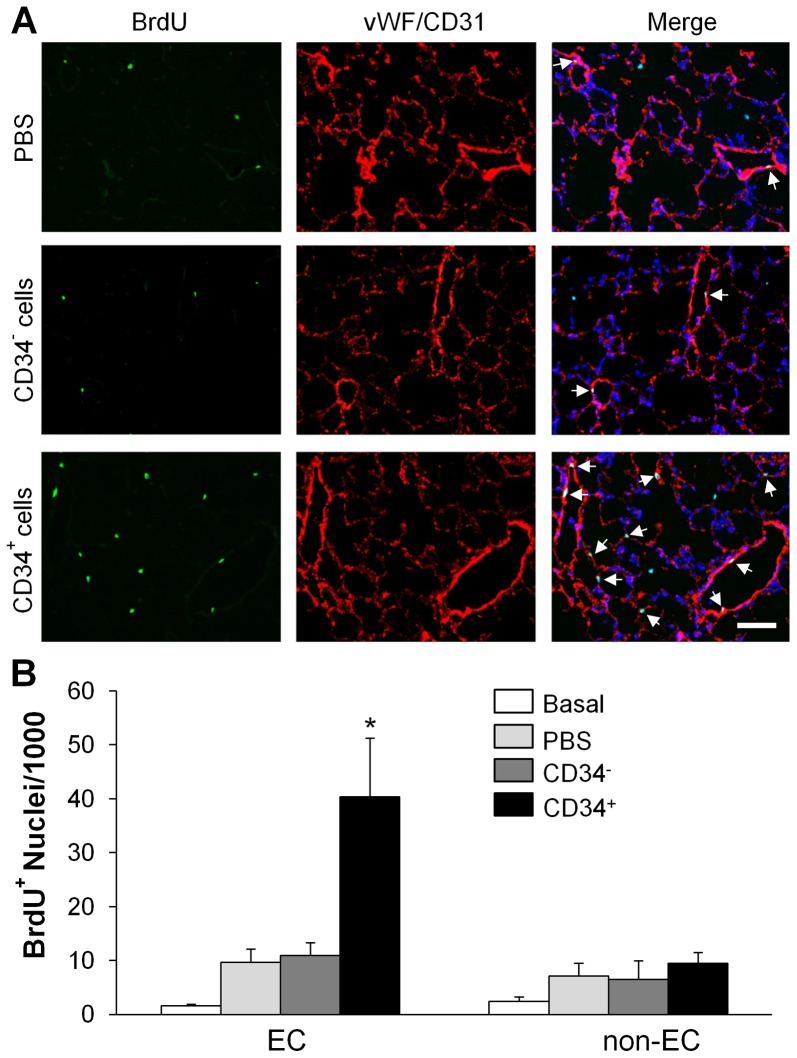
fCB-CD34^+^ cell treatment-induced endothelial cell proliferation in lungs. (**A**) Representative micrographs of immunofluorescent staining. Lung tissues were collected at 52 h post-LPS challenge, sectioned and immunostained with anti-BrdU (green) and anti-vWF/CD31 (red) antibodies. Nuclei were counterstained with DAPI (blue). Arrows indicate proliferating EC. Scale bar: 50 µm. (**B**) Quantification of BrdU-positive EC (vWF^+^ and/or CD31^002B^) and non-EC (vWF^−^ and/or CD31^−^). Data are expressed as mean ± SD (*n* = 4/group). *, *P*<0.001 versus PBS or CD34^−^.

To determine whether fCB-CD34^+^ cell treatment induces endothelial cell proliferation thereby promotes vascular repair [31], we assessed cell proliferation by *in vivo* BrdU labeling. Quantification of BrdU-positive EC (expressing either CD31 mainly in large vessels or vWF in capillaries) revealed that fCB-CD34^+^ cell treatment induced a marked increase of EC proliferation in mouse lungs at 52 h post-LPS challenge which was approximately 4-fold greater than PBS-treated controls. EC proliferation in lungs of mice treated with CD34^−^ was similar to PBS-treated controls (**Fig. 6**).

## Discussion

In this study, we have investigated the effects of intravenous administration of freshly isolated human umbilical cord blood CD34^+^ progenitor cells on LPS-induced lung injury in mice. We observed that fCB-CD34^+^ cell treatment inhibited lung vascular injury and inflammation following LPS challenge. The mice treated with fCB-CD34^+^ cells exhibited a marked increase of survival rate. We also observed a significant increase of endothelial proliferation in lungs of mice treated with fCB-CD34^+^ cells which is associated with rapid restoration of vascular integrity and inhibition of lung inflammation. Taken together, these results show that freshly isolated human CD34^+^ progenitor cells exert protective effects in mice against inflammatory lung injury induced by LPS and also promote vascular repair through induction of endothelial regeneration.

To our knowledge, this is the first study to demonstrate that freshly isolated human cord blood-derived CD34^+^ cells are protective against inflamamtory lung injury induced by LPS challenge. Previous study has shown these cells promote regeneration of injured alveolar epithelium in models of neonatal lung injury/bronchopulmonary dysplasis [35]. Other studies using cultured human umbilical cord-derived progenitor cells have also demonstrated the beneficial effects of these in vitro-expanded cells in various lung disease models such as neonatal hyperoxic lung injury [36], [37], bleomycin-induced lung injury [38], and E. coli-induced acute lung injury [39]. Mao, et.al. has shown that in vitro-expanded cord-blood-derived CD34^+^ cells but not freshly isolated CD34^+^ cells induce lung growth and alveolarization in injured newborn lungs [40], suggesting the cultured CD34^+^ cells express a unique set of factors required for lung growth and alveolarization. However, our study shows that freshly isolated CD34^+^ cells are protective against LPS-induced inflammatory lung injury. The discrepancy is likely ascribed to different animal models and different aspects of protective effects. These studies suggest that inhibition of inflammatory lung injury and promotion of vascular repair is mediated by mechanisms different from lung growth and alveolarization.

Persistently increased lung microvascular permeability resulting in protein-rich lung edema is a hallmark of ALI/ARDS [1]–[4]. Thus, targeting microvascular leakiness to restore lung fluid homeostasis is a potential therapeutic approach for the prevention and treatment of ALI/ARDS. Our data have shown that fCB-CD34^+^ cell treatment resulted in a marked decrease of lung vascular permeability as determined by both *K*_f,c_ measurement and EBA extravasation assay. Consistently, our previous studies using *in vitro* expanded mouse bone marrow-derived progenitor cells (approximately 80% of them are CD34^+^) also show similar inhibitory effects on vascular permeability induced by LPS and thrombin challenge [41], [42]. Other studies with human MSC have shown decreased lung edema formation and lung vascular and epithelial barrier permeability in various animal model of ALI [5]–[8]. These data suggest human fCB-CD34^+^ cells exhibit similar vascular protective effects as other adult stem cells.

The well-known effects of human MSC are their immunomodulatory and anti-inflammatory effects. It has been reported that human MSC can negatively regulate the functions of macrophages and exert anti-inflammatory effect by secretion of paracrine factors. For example, in a mouse model of ALI induced by LPS, the administrations of MSC induce more production of IL-10 and then ameliorate lung inflammation, which is eliminated by macrophage depletion or pretreatment with IL-10 antibodies [11]. Many studies have shown that MSC treatment markedly inhibits expression of proinflammatory molecules and lung inflammation [5]–[11]. Our data have shown that human fCB-CD34^+^ cells but not CD34^−^ cells inhibit lung inflammation evident by decreased lung MPO activity and neutrophil infiltration in the lung parenchyma. We also observed drastic inhibition of expression of proinflammatory molecules including TNF-α, IL-6, ICAM-1 and iNOS. Collectively these data suggest human fCB-CD34^+^ cells are potent immunomodulators and have strong anti-inflammatory effects.

As seen in the acute phase of ALI in humans [1]–[4], endothelial injury induced by LPS is characterized by increased endothelial permeability [43], [44]. Administration of hfCB-CD34+ cells markedly induce endothelial cell proliferation which is an important component of endothelial regeneration following lung vascular injury [31], [33], [45]. Induced endothelial cell proliferation results in rapid restoration of vascular integrity and inhibition of lung inflammation. Consistently, lung inflammation as indicated by MPO activity and neutrophil sequenstration in fCB-CD34^+^-treated mice is returned to basal level as quickly as 52 h post-LPS challenge whereas prominent lung inflammation is still evident at the same time period in control mice treated with either PBS or CD34^−^ cells. It has been shown that MSC secrete an array of angiogenic groth factors, including keratinocyte growth factor, and vascular endothelial growth factor [13], [46]. We predict that fCB-Cd34+ cells also secrete similar angiogenic factors and thereby induce endothelial cell proliferation and restore vascular integrity. Future study is warranted to determine which angiogenic factor(s) are released by fCB-CD34+ cells and the endogenous mediator(s) responsible for these growth factor(s)-induced endothelial cell proliferation.

The promising role of human MSC in stem cell-based therapies appears to be limited due to a decline of their regenerative potential with increasing donor age, and the need of in vitro expansion prior to treatment. Accumulating evidence has shown the properties and behaviors of human MSC may change during cultivation. Thus the therapeutic effects of the MSC cannot rule out the participation of factors in the medium during cultivation. Recent studies demonstrated that cultivation may reduce MSC adhesion to laminin and endothelium [47]. During in vitro expansion, MSC progressively lose their progenitor characteristics [48], increase number of chromosomal abnormalities [49], [50], and compromise mitochondrial function [51], suggesting *in vitr*o cultivation may affect the character and effects of human adult stem cells. Thus, fresh isolation without *in vitro* expansion excludes the potential artificial effects from cultivation or other supplements in the medium. Additionally, it is a generalized concept that MSC function mainly through paracrine mechanisms. It is unclear whether some of these paracrine effects are induced by *in vitro* culture. In this study we have tested the effects of freshly isolated human CD34^+^ cells without *in vitro* culture on LPS-induced acute lung injury. As seen with MSC, we have shown similar therapeutic effects of these freshly isolated cells, suggesting that the paracrine factors are intrinsic factors of adult stem cells by which these cells exert their therapeutic potential.

In conclusion, our study has demonstrated the great potential of freshly isolated human cord blood CD34^+^ progenitor cells on the treatment of ALI/ARDS. Given the easy availability, rapid purification procedure without defects from *in vitro* culture, and low immunogenicity of these cells [52] as well as the nature of rapid progression of ALI/ARDS, fCB-CD34+ cells may be an important source of stem cells for the prevention and treatment of ALI/ARDS.
